# The effectiveness of mapping-targeted biopsies on the index lesion in transperineal prostate biopsies

**DOI:** 10.1590/S1677-5538.IBJU.2023.0558

**Published:** 2024-03-10

**Authors:** Nahuel Paesano, Violeta Catalá, Larisa Tcholakian, Xavier Alomar, Miguel Barranco, Enric Trilla, Juan Morote

**Affiliations:** 1 Creu Blanca Clinic Barcelona Catalunya Spain Creu Blanca Clinic Barcelona, Catalunya, Spain;; 2 Vall d'Hebron University Hospital Barcelona Department of Urology Catalunya Spain Department of Urology Vall d'Hebron University Hospital Barcelona, Catalunya, Spain;; 3 Universitat Autonoma de Barcelona Department of Surgery Barcelona Catalunya Spain Department of Surgery, Universitat Autonoma de Barcelona, Barcelona, Catalunya, Spain

**Keywords:** Prostatic Neoplasms, Prostate, Retrospective Studies

## Abstract

**Purpose:**

To evaluate the effectiveness of mapping-targeted biopsies (MTB) on the index lesion for the detection of clinically significant prostate cancer (csPCa) in transperineal fusion-image prostate biopsies.

**Materials and Methods:**

A retrospective analysis was conducted on 309 men with suspected PCa who underwent prostate biopsies at the Creu Blanca reference center in Barcelona, Spain. The Prostate Imaging-Reporting and Data System (PI-RADS v.2.1) of the magnetic resonance images (MRI) were reclassified by an expert radiologist reading of pre-biopsy biparametric MRI used for segmentation of suspected lesions. Transperineal MTB of suspicious lesions and 12-core systematic biopsies were performed using the Artemis™ platform. CsPCa was defined as International Society of Urological Pathology grade group ≥ 2.

**Results:**

CsPCa was detected in 192 men (62.1%), with detection rates of 6.3% for PI-RADS 2, 26.8% for PI-RADS 3, 87.3% for PI-RADS 4, and 93.1% for PI-RADS 5. MTB of the index lesion identified 185 csPCa (96.3%). CsPCa was detected solely in systematic biopsies in three cases (1.6%), while an additional four cases (2.1%) were identified only in the second suspected lesion. A predictive model for csPCa detection in MTB of the index lesion was developed, with an AUC of 0.918 (95% CI 0.887-0.950).

**Conclusions:**

This model had the potential to avoid 23.3% of prostate biopsies without missing additional csPCa cases. MTB of the index lesion was highly effective for identifying csPCa in fusion transperineal prostate biopsies. A developed predictive model successfully reduced the need for almost one quarter of biopsies without missing csPCa cases.

## INTRODUCTION

Prostate cancer (PCa) currently stands as the most prevalent malignancy and the third leading cause of cancer-related deaths among men in both the United States (US) and the European Union (EU) (1, 2). PCa screening has remained controversial due to the adverse consequences of unnecessary prostate biopsies and the over-detection of insignificant tumors (iPCa) (3). Clinically significant PCa (csPCa) in prostate biopsies can have various definitions. It is most defined when the current grade groups of the International Society of Urologic Pathology (ISUP-GG) are grade 2 or higher (4). In recent years, additional tools have been proposed to mitigate the need for unnecessary biopsies and to reduce the over-detection of iPCa. These include new tumor markers, predictive models, and the traditional prostate-specific antigen density (PSA D). However, the most significant contribution has arisen from prostate magnetic resonance imaging (MRI) and the possibility of performing targeted biopsies of suspicious areas (5).

The implementation of multiparametric MRI (mpMRI) and the enhanced interpretation provided by the Prostate Imaging-Reporting and Data System (PI-RADS) have been essential in identifying suspicious csPCa and assessing its semi-quantitative risk (6, 7). These innovations, coupled with the evolution of PI-RADS for categorizing prostate lesions on a scale ranging from very low to high suspicion of harboring csPCa, have prompted the development of software solutions that facilitate the fusion of MRI-observed areas with real-time transrectal ultrasound (TRUS) images (8).

Image-fusion prostate biopsy is an effective procedure that not only enhances the detection of csPCa but also reduces the likelihood of over-detecting iPCa, regardless of the transrectal or transperineal route (9). The transrectal approach has been the most used method for prostate biopsies on a global scale for many decades (10). However, in 2019, the European Association of Urology (EAU) PCa guidelines made the primary recommendation for transperineal biopsies over transrectal biopsies based on a meta-analysis of seven studies involving 1,330 biopsied men. This analysis demonstrated a significant reduction in infective complications associated with the transperineal approach compared to transrectal biopsies (11).

The fusion software platforms allow targeted biopsies of suspicious areas previously identified by mpMRI. These biopsies are usually combined with systematic biopsies in the rest of the prostate gland. In cases where an MRI reveals more than one PI-RADS lesion >3, the lesion with the highest degree of aggressiveness according to PI-RADS is considered the index lesion, or the largest lesion in the case of two lesions with the same PI-RADS score (12).

The complementarity of systematic and targeted biopsies, defined as the amount of csPCa detected only in one of them, can be attributed to multifocal csPCa, MRI-invisible lesions, errors in lesion targeting, and MRI lesions being missed by radiologists (13). The complementarity of targeted and systematic biopsies has been analyzed using the transrectal approach in a systematic review by the Cochrane organization (14), and currently, the EUA recommends performing both types of biopsies (11).

Due to the current trend of reducing the aggressiveness of prostate biopsies to decrease procedure complications and overdiagnosis of iPCa, it is crucial to continue generating evidence on the effectiveness of targeted transperineal biopsies with image fusion (15). The optimal biopsy strategy, in terms of spatial distribution and the number of cores required, has not yet been clearly defined. Specifically, at least eight systematic biopsy cores should be taken bilaterally, depending on prostate volume, while three to five biopsy cores guided by magnetic resonance imaging are needed to compensate for the risk of target imprecision (11, 16, 17). Conversely, recent studies suggested that distant systematic biopsy cores in relation to the index lesion on magnetic resonance imaging play a limited role in detecting csPCa (18).

We hypothesize that prostate biopsies from the index lesion are sufficient to appropriately diagnose clinically significant prostate cancer. Our objective is to assess the efficacy of exclusively utilizing targeted biopsies by mapping the index lesion in the detection of clinically significant prostate cancer, and to evaluate the effectiveness of index biopsies compared to systematic biopsies through the transperineal approach.

## MATERIAL AND METHODS

### Design, Setting, and Participants

This retrospective analysis relates to the first-year prospective trial aimed at validating the Barcelona risk calculators (BCN-RCs) (19, 20). The study was conducted at a reference center specializing in transperineal MRI-TRUS fusion prostate biopsy, located at Creu Blanca in Barcelona, Spain. From January 1 to December 31, 2022, a total of 309 consecutive men with suspected prostate cancer—defined by a serum PSA level >3.0 ng/mL and/or an abnormal digital rectal examination (DRE), along with a positive mpMRI (PI-RADS ≥ 3)—were referred for prostate biopsy. Prostate biopsies in PI-RADS 2 lesions, are not typically referred for biopsy. However, prostate biopsies of PI-RADS 2 lesions were performed in those patients at high risk of CsPCa, for example, if the PSAD is >0.15. The study was executed in accordance with the Standards of Reporting for MRI-Targeted Biopsy Studies (START) guidelines (21). The analysis was performed using an anonymized database, and the project received approval from the Vall d'Hebron Ethics Committee (PRAG-02/2020). Furthermore, it was supported by the Instituto de Salut Carlos III of Spain (PI20/01666).

### MRI Image Acquisition Data and Segmentation

The studies conducted at the Creu Blanca center were carried out using a Siemens Verio™ 3 Tesla MRI Scanner (Siemens Inc, Munich, Germany) equipped with a pelvic-phased array. The mpMRI protocol included transversal sections in SET1 and TSG-SET2 sequences, as well as coronal and sagittal TSET2 sequences in both 2D and 3D. Diffusion studies (ADC and DWI calculated at 1600-2000-3000) and perfusion during contrast injection were also performed. Lesions were reported in accordance with PI-RADS v.2.1 (22).

For patients referred with mpMRIs performed at other centers, an expert radiologist reclassified the lesions to PI-RADS v.2.1 based on a biparametric MRI (bpMRI) conducted for lesion segmentation. All studies adhered to the criteria established by the European Society of Uro-radiology (ESUR) (23). Up to three suspicious lesions were included. Segmentation of suspicious lesions was achieved using the ProFuse™ semi-automatic system (Eigen, California, USA). Suspected PCa men with reclassified lesions to PI-RADS score 2 received targeted biopsies of these lesions.

### Transperineal prostate biopsy

All prostate biopsies were performed transperineally in the lithotomy position under general anesthesia by four urologists with extensive experience in this technique. MRI-TRUS image fusion was performed with the ARTEMIS™ robotic system (Eigen, California, USA). Core biopsy samples were obtained using the BARD Magnum™ gun with 18-gauge x 20-cm prostate biopsy needles (Bard Care, Crawley, UK) under ultrasound vision (HITACHI ALOKA NOBLUS™, HITACHI, Connecticut, USA).

**Figure 1:**
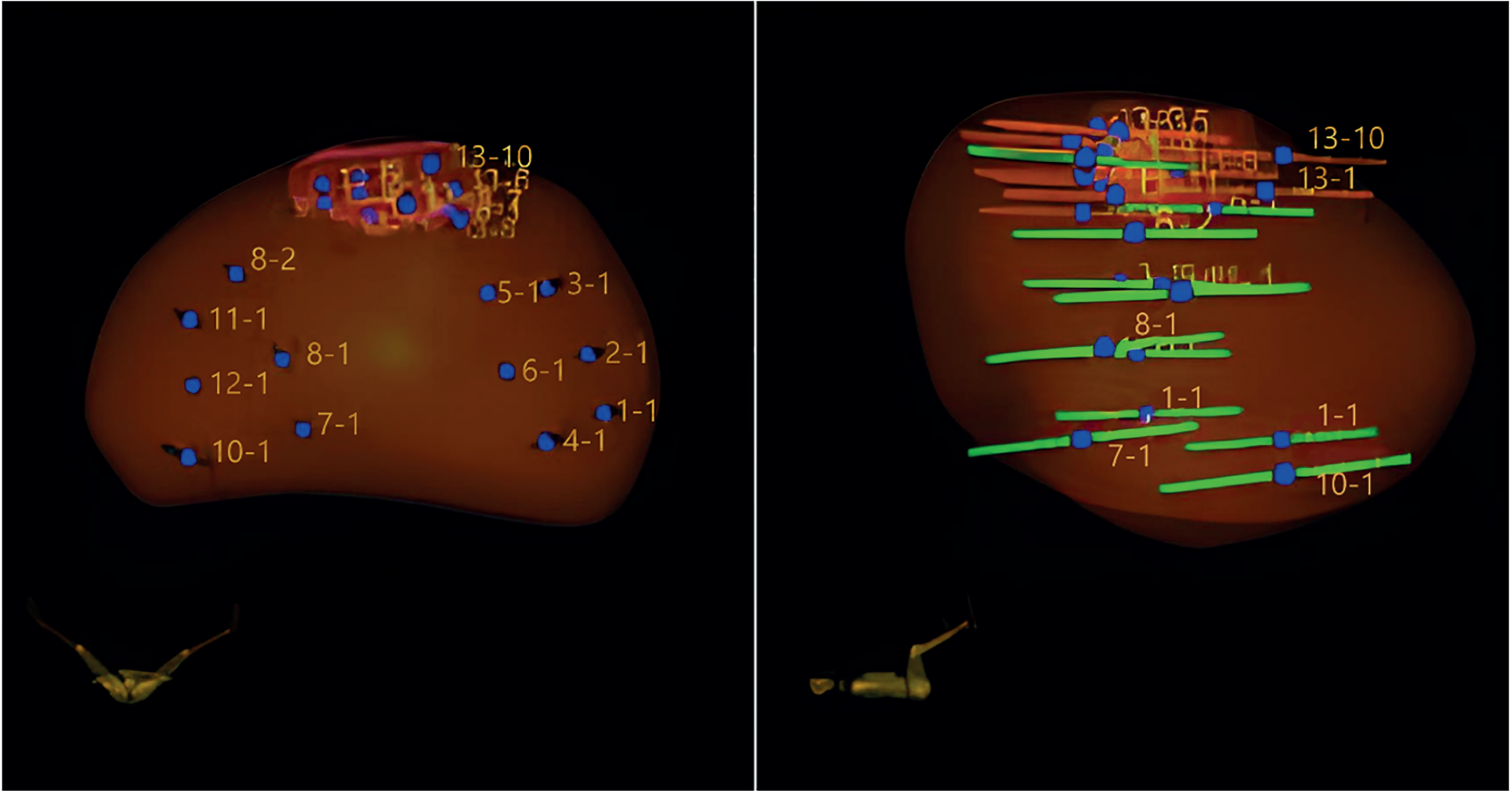
Biopsy method. Cores were obtained from a systematic template and MRI lesion.

The prostate biopsy protocol included first obtaining mapping-targeted biopsies of a maximum of three suspicious areas by 0.5-mm mapping of each lesion and perilesional area. Thereafter, a systematic biopsy was performed to obtain 12 cores according to the scheme suggested by the ARTEMIS™ platform, excluding the suspicious areas. Each core of the systematic biopsy was numbered and analyzed as an independent sample (1 to 12). The cores of each suspected area were grouped as samples 13 to 15. The mapping-targeted biopsies scheme used is illustrated in Figure-1.

### Histopathological diagnosis

A urologic pathologist with more than 10 years of experience in prostatic pathology analyzed the obtained material. The pathology report considered the number of positive cores, length of tumor, and International Society of Urologic Pathology (ISUP) grade group. iPCa was defined with an ISUP-GG of 1, while csPCa was considered when the ISUP-GG was 2 or higher (4, 24).

### Variables in the study

Age (years), PCa family history (first degree), type of biopsy (initial vs. repeated), DRE (normal vs. suspicious), DRE prostate volume category (small, medium, large), PSA (ng/mL), MRI prostate volume, the number of suspicious areas, PI-RADS v2.1 score, maximum diameter of lesions (mm), site of lesions (right vs. left), and location (peripheral base-center, peripheral apex, central-transition zone, or anterior zone). Pathology findings were reported as follows: ISUP grade group (GG) of tumors detected and its length in each core affected, the total number of cores submitted, and positive samples according to their location in the systematic biopsy and mapping-targeted biopsies.

### Endpoint variables

The endpoint variable was the detection of csPCa.

## Statistical Analysis

Quantitative variables were described as medians and interquartile ranges (IQRs: 25th–75th percentile). Qualitative variables were described as percentages. The association between qualitative variables was analyzed using Pearson's chi-square test. The concordance between systematic and targeted biopsies was assessed using the Kappa index. Independent predictive variables for csPCa detection in mapping-targeted biopsies of the index lesion were identified using binary logistic regression. A predictive model for csPCa detection was developed, and probabilities of csPCa were calculated. The discrimination ability of the model was assessed using receiver operating characteristic (ROC) analysis, and the area under the curve (AUC) and 95% confidence interval were calculated. The net benefit of the model over biopsying all men was analyzed using decision curve analysis (DCA), and specificities corresponding to 100%, 97.5%, and 95% sensitivity thresholds were analyzed. Significant differences were considered when the p values were less than 0.05. Statistical analysis was performed using SPSS v.25 (IBM Corp., Armonk, NY, USA).

## RESULTS

The characteristics of the 309 men included in this study are shown in Table-1. The median age at biopsy was 67 years (61-72) with a median serum PSA level of 6.3 ng/mL (4.8-9.3). The median prostate volume, as reported from MRI, was 53 cc (39-75). The median PSA density was 0.14 ng/mL/cc. In 52 men (16.8%), a family history of PCa existed, and 78 men (25.2%) had undergone at least one previous negative biopsy. The digital rectal examination (DRE) was suspicious in 47 men (15.2%). In 223 cases, only one suspicious lesion existed (72.2%), in 75 (24.2%), two, and in 11 cases, three (3.6%). After the biparametric MRI expert reading of the PI-RADS v.2.1, the index lesion was classified as 2 in 48 cases (15.5%), 3 in 71 (23%), 4 in 118 (38.2%), and 5 in 72 (23.3%). Within the second lesions, 5 (6.7%) were PI-RADS 2, 23 (30.7%) PI-RADS 3, 41 (54.7%) PI-RADS 4, and 6 (8.0%) PI-RADS 5. Among the 11 third lesions, 4 (36.4%) were PI-RADS 3, and 7 (63.6%) were PI-RADS 4. A median of 22 total cores (15-30) were obtained from mapping-targeted biopsies of suspicious and systematic biopsies. PCa was detected in 230 men (74.4%), with 192 (62.1%) identified as csPCa, and 38 (12.3%) as iPCa.

**Table 1:** Suspected PCa Men Characteristics.

Characteristic		Measurement
Number of men		309
Median age (IQR), years		67 (61-72)
Median PSA (IQR), ng/mL		6.3 (4.8-9.3)
Suspicious DRE, n (%)		47 (15.2)
Repeated biopsy, n (%)		78 (25.2)
PCa family history, (%)		52 (16.8)
Median prostate volume (IQR), cc		53 (39-75)
**mpMRI lesions, n (%)**		
	1	309 (100)
	2	75 (24.2)
	3	11 (3.6)
**PI-RADS of lesion 1 (index), n (%)**		**309**
	2	48 (15.5)
	3	71 (23.0)
	4	118 (38.2)
	5	72 (23.3)
**PI-RADS of lesion 2, n (%)**		**75**
	2	5 (6.7)
	3	23 (30.7)
	4	41 (54.7)
	5	6 (8.0)
**PI-RADS of lesion 3, n (%)**		**11**
	3	3 (36.4)
	4	7 (63.6)
PCa detection, n (%)		230 (74.4)
csPCa detection, n (%)		192 (62.1)
iPCa, n (%)		38 (12.3)
**csPCa-iPCa detection according to index lesion PI-RADS**		
	2, n (%)	3 (6.3) - 7 (14.6)
	3, n (%)	19 (26.8) - 19 (26.8)
	4, n (%)	103 (87.3) - 7 (5.9)
	5, n (%)	67 (93.1) - 5 (6.9)

**IQR** = interquartile range; **PSA** = prostate-specific antigen; **DRE** = digital rectal examination; **PCa** = prostate cancer; **PI-RADS** = Prostate imaging-report and data system; **csPCa** =clinically significant PCa; **iPCa** = insignificant PCa

The distribution rates of csPCa and iPCa according to the PI-RADS v.2.1 categories are shown in Table-1. The csPCa detection rate was 6.3% in PI-RADS 2, 26.8% in PI-RADS 3, 87.3% in PI-RADS 4, and 93.1% in PI-RADS 5 (p < 0.001). The iPCa over-detection rates were 14.6% in PI-RADS 2, 26.8% in PI-RADS 3, 5.9% in PI-RADS 4, and 6.9% in PI-RADS 5 (p <0.001).

**Figure 2:**
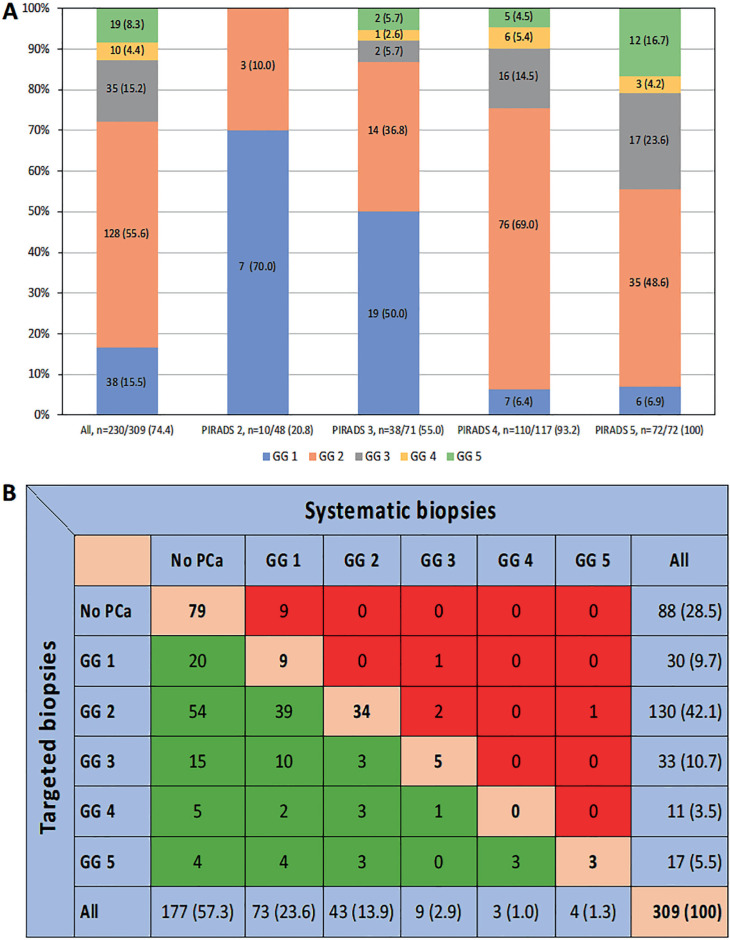
Distribution of grade groups in all PCa detected and according to the PI-RADS category of the index lesion (A), and correlation of biopsy findings (from no PCa to the highest-grade group) observed in targeted and systematic biopsies (B).

The distribution of ISUP-GGs in all PCa detected according to the PI-RADS categories in the index lesion is presented in Figure-2A The over-detection of iPCa decreased, and the detection of csPCa increased as the PI-RADS category became higher (p < 0.001). The correlation between the ISUP-GG of PCa detected in targeted and systematic biopsies is presented in Figure-2B We observed that 9 ISUP-GG 1 cases were detected in the systematic biopsies in men without PCa detected in targeted biopsies. One ISUP-GG 3 case was detected in the systematic biopsies performed in men with ISUP-GG 1 in targeted biopsies. We found 3 cases in which systematic biopsies upgraded the ISUP-GG 2 detected in targeted biopsies (2 cases to GG 3 and 1 to GG 5). No cases with ISUP-GG 3 and 4 in targeted biopsies upgraded in systematic biopsies. In summary, 1 of 30 iPCa cases upgraded to csPCa (3.3%), while 3 of 130 ISUP-GG 2 cases (2.3%) upgraded to GG 3 (2) and GG 5 (1).

CsPCa was detected in both targeted and systematic biopsies in 56 cases (18.1%), solely in targeted biopsies in 133 (43.0%), and only in systematic biopsies in three cases (1.0%) (p < 0.001, and Kappa index 0.239). Targeted biopsies of the index lesion identified 185 of the 189 csPCa cases detected within all targeted biopsies (97.9%). Four csPCa cases were additionally detected in the targeted biopsy of the second suspicious lesion (2.1%), while the targeted biopsies of 11 third suspicious lesions did not detect any additional csPCa case (0%). The ISUP-GGs of PCa detected in the index lesion and those additionally detected in the second targeted biopsy are reported in Table-2.

**Table 2:** CsPCa Detection in Targeted Biopsies. New csPCa Detected Only in Secondary Lesions and its grade group.

Lesion	Number of lesions, n	csPCa n (%)*	New csPCa, n (%)#	Grade group of csPCa			
				2	3	4	5
Index	309	185 (59.9)	185 (97.9)	125	33	9	18
Second	75	40 (53.3)	4 (2.1)	4⊥	0	0	0
Third	11	2 (18.2)	0 (0)	0	0	0	0
All	395	227 (100)	189 (100)	131	35	9	17

n = number; csPCa = clinically significant prostate cancer;

* percent referred to the number of lesions;

# percent referred to all csPCa detected;

⊥ Three men have GG 1 in the index lesion while one had no PCa.

In terms of core samples, if targeted biopsies of the index lesion were solely performed, the total number of core samples obtained, which was 6,573, would be reduced to 2,392 (63.6%). This reduction would result from 3,708 (56.4%) core samples obtained in systematic biopsies, 426 (6.5%) in the second suspicious lesion, and 47 (0.7%) in the third suspicious lesion.

To investigate the development of a predictive model for the detection of clinically significant prostate cancer (csPCa) in mapping-targeted biopsies of the index lesion, we conducted a logistic regression analysis involving 12 independent predictive variables related to the characteristics of the patients, MRI findings, and the number of core samples obtained. Serum PSA, with an odds ratio (OR) of 1.136 (95% confidence interval: 1.010-1.277, p = 0.033), prostate volume, with an OR of 0.987 (95% confidence interval: 0.365-0.991, p < 0.001), and the PI-RADS score, with an OR of 7.285 (95% confidence interval: 4.455-11.913, p < 0.001), emerged as the independent predictors of csPCa, as presented in Table-3.

**Table 3:** Multivariate Analysis Searching Independent predictors for csPCa detection.

Candidate predictors	Odds ratio (95% CI)	p Value
Age, Ref. one year	1.039 (0.991-1.090)	= 0.101
PCa family history, Ref. no	2.013 (0.761-5.324)	= 0.159
Repeated biopsy, Ref. no	1.649 (0.734-3.758)	= 0.234
Serum PSA, Ref. one ng/mL	1.136 (1.010-1.277)	= 0.033
DRE, Ref. normal	1.094 (0.354-0.381)	= 0.875
Prostate volume, Ref one cc	0.978 (0.365-0.991)	< 0.001
Number of MRI lesions, Ref. 1	2.212 (0.848-5.160)	= 0.066
PI-RADS score of index lesion, Ref. 2	7.285 (4.455-11.913)	< 0.001
Diameter of index lesion, Ref. one mm	1.005 (0.930-1.096)	= 0.901
Number of targeted cores to index lesion, Ref. 2 cores2	1.167 (0.993-1.372)	= 0.061
Zone of lesion, Ref. CZ	0.710 (0.265-1.899)	= 0.495
Position of index lesion, Ref. posterior	0.730 (0.255-2.090)	= 0.557

CI = confidence Interval; PSA = prostate-specific antigen; MRI = magnetic resonance imaging; PI-RADS = prostate imaging-report and data system; CZ = central zone.

**Figure 3:**
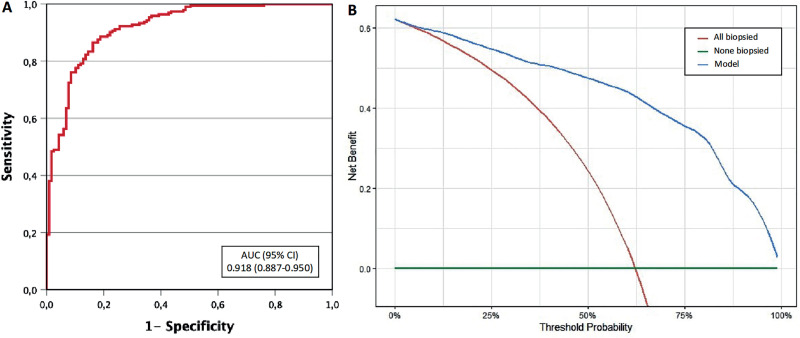
Discrimination ability for csPCa of the developed model based for performing only targeted biopsy of the index lesion (A), and decision curve analysis showing the benefit of the developed model compared to conduct targeted biopsies of the index lesion in all cases (B).

The ROC curve of the developed predictive model demonstrated an AUC of 0.918 (95% confidence interval: 0.887-0.950), illustrated in Figure 3A. Application of the model revealed a net benefit over the strategy of performing biopsies on all men, as shown in Figure 3B. Specificities were provided for sensitivity thresholds of 100%, 97.5%, and 95%, with values of 239, 52.1, and 64.1%, respectively. Using the 100% sensitivity threshold, it would be possible to avoid 72 (23.3%) mapping-targeted biopsies of the index lesion without missing additional cases of csPCa, beyond the six cases (3.1%) that were only detected in systematic biopsies and targeted biopsies of the second suspicious lesions (note that one case was detected in both types of biopsies).

Following the implementation of the developed model, 602 additional core samples, corresponding to the 72 avoided mapping-targeted prostate biopsies of the index lesion, would be spared. When combined with the previously estimated 63.6% reduction in core samples resulting from exclusively performing mapping-targeted biopsies of index lesions, this would result in a total reduction of 72.8% in core samples.

## DISCUSSION

Early detection of csPCa continues to improve with the implementation of new MRI-TRUS image fusion software for performing targeted biopsies (25), new strategies for biopsying (26), and the recent recommendation for the transperineal approach (27). The challenge is to detect the maximum amount of csPCa while minimizing the over-detection of iPCa and secondary side effects (9, 28).

In this study, we report, to the best of our knowledge, the first series of MRI-TRUS fusion image biopsies with the Artemis™ platform through the transperineal route, utilizing mapping-targeted biopsies of suspicious lesions and a 12-core systematic biopsy. The overall PCa detection rate reached 74.4%, with 62.1% for csPCa and 12.3% for iPCa. These detection rates of csPCa and iPCa appear to be well balanced with the expectations in PI-RADS v.2.1 and are appropriate when compared with other series (29).

In the current context of precision medicine, the multifocal nature of PCa poses a significant challenge (30). In the quest to determine the best protocol for performing targeted prostate biopsies using fusion images from MRI for the most accurate diagnosis and the lowest aggressiveness, several series have been published with multiple biopsy protocols. These include sampling only in the peripheral prostate area, avoiding transition zone biopsy (31), performing saturation biopsies in the target area (32), combine PSA D and PI-RADS to exclusively conduct target biopsies in patients with elevated PSA D, eliminating the need for systematic biopsies (33), using Umbra (targeted lesion) and Penumbra (perilesional) samples (34), optimizing the number of cores by obtaining only three or fewer core samples per targeted lesion (35), or conducting only biopsy samples in the target lesion without systematic biopsies (36).

However, the additional value of MRI-guided biopsies of suspicious secondary lesions and systematic biopsies over index lesion mapping-guided biopsies for the detection of csPCa in fusion transperineal prostate biopsies is not yet fully established. Rachubinski et al. (37) evaluated the diagnosis of csPCa in index lesions and secondary lesions of 571 patients who underwent prostate biopsy using transperineal image fusion. This study concluded that targeted biopsy of the secondary lesion can provide valuable diagnostic information in most clinical scenarios and should not be omitted in most fusion biopsy protocols. Previous prospective studies and a meta-analysis indicate an added value of systematic biopsy for detecting csPCa, with rates ranging between 4.3% and 5.2% for the ISUP grade 2 group and between 1.2% and 4.1% for the ISUP grade 3 group in patients without prior biopsy (38, 39). Our study showed that mapping-targeted biopsies of the index lesion identified 96.3% of csPCa, and it was necessary to perform mapping-targeted biopsies of the secondary lesion to detect an additional 2.1% of csPCa cases. Another 1.6% of csPCa was only detected in systematic biopsies (see below). Our findings also revealed that biopsy at the third lesion did not identify any new cases of csPCa.

Regarding the complementarity of systematic biopsies for detecting csPCa, Petov et al. (40) have published a meta-analysis concluding that targeted and systematic biopsies have comparable csPCa detection rates. Marra et al. (41) reported that the PCa detection rate of targeted biopsy was higher than that of systematic biopsies, but their role should not be overlooked, and the combination of targeted and systematic biopsies is essential. In line with the results of these studies, the Cochrane systematic review makes its position clear regarding the complementarity of systematic biopsies with targeted biopsies via the transrectal route (14). However, the complementarity of both types of biopsies via the transperineal route is not yet well established. Porpiglia et al. (42) compared the detection rate of csPCa between targeted biopsy alone and targeted biopsy complemented with systematic biopsy. This trial was designed as a non-inferiority study and concluded that fusion-targeted biopsy alone was not inferior to the fusion biopsy complemented with systematic biopsy for the detection of csPCa by the transperineal approach. In our study, the complementarity analysis between mapping-targeted biopsy and systematic biopsy showed that csPCa was only detected in systematic biopsy in three cases (1%). Regarding the correlation between mapping-targeted biopsies and systematic biopsies, we observed that nine ISUP-GG 1 tumors were detected in the systematic biopsies in men with no PCa detected in targeted biopsies, which increased the rate of over-detection. In three cases, systematic biopsies did not yield any new diagnosis of csPCa but resulted in an up-grading of targeted biopsies from ISUP-GG 2 to ISUP-GG 3 in two cases, and to ISUP-GG 5 in one case. We consider these findings important for establishing the management of localized PCa with active surveillance or focal therapy (43).

Having established the high effectiveness of mapping-targeted biopsy of the index lesion, identifying 96.3% of csPCa, we developed a new predictive model for detecting csPCa in mapping-targeted biopsies of the index lesion. This model reported a high discrimination ability, allowing us to avoid almost one quarter of prostate biopsies without missing additional csPCa, accounting for seven cases (3.6%); of these, four were detected only in the second suspicious lesion, and three only in the systematic biopsy. Additionally, to reduce almost one quarter of prostate biopsies, the application of this model decreased the aggressiveness of the overall prostate biopsy approach. In summary, the strategy of performing only mapping-targeted biopsy of the index lesion and applying the developed predictive model avoided almost one quarter of prostate biopsies and reduced the aggressiveness of overall prostate biopsies by reducing 72.8% of the 6,573 core samples obtained.

Our study is affected by several limitations, primarily due to its retrospective and non-randomized design. This study was conducted under a specific biopsy protocol, using the Artemis™ platform in a single center. An expert reading of MRI, reclassifying PI-RADS v.2.1, accurate segmentation of suspicious lesions by an expert radiologist, and a limited number of experienced surgeons performing the procedure may have contributed to the high rate of csPCa detection. Future well-designed studies will be needed to analyze simple and efficient strategies for prostate biopsy while minimizing its aggressiveness.

## CONCLUSIONS

Transperineal mapping-targeted biopsy of the index lesion was effective in identifying over 95% of csPCa. Furthermore, the application of a developed predictive model for csPCa detection in mapping-targeted biopsy of the index lesion reduced the number of prostate biopsies by almost one quarter without missing additional csPCa. This combined strategy significantly decreased the aggressiveness of prostate biopsies by reducing almost 75% of core samples obtained, particularly when using the Artemis™ robotic platform.
